# Bacteriocin production by mucosal bacteria in current and previous colorectal neoplasia

**DOI:** 10.1186/s12885-020-6512-5

**Published:** 2020-01-16

**Authors:** Darina Kohoutova, Miroslava Forstlova, Paula Moravkova, Jiri Cyrany, Juraj Bosak, David Smajs, Stanislav Rejchrt, Jan Bures

**Affiliations:** 1 2nd Department of Internal Medicine Gastroenterology, Charles University, Faculty of Medicine in Hradec Kralove, University Hospital, Sokolska 581, 500 05 Hradec Kralove, Czech Republic; 2 The Royal Marsden Hospital NHS Foundation Trust, Fulham Road, Chelsea, London, SW3 6JJ UK; 3 Department of Clinical Microbiology, Charles University, Faculty of Medicine in Hradec Kralove, University Hospital, Sokolska 581, 500 05 Hradec Kralove, Czech Republic; 4 Department of Biology, Masaryk University, Faculty of Medicine, University Campus at Bohunice, Kamenice 753/5, 625 00 Brno, Czech Republic

**Keywords:** Gramnegative bacteria, Colicin, Microcin, Colorectal neoplasia, Colorectal carcinoma

## Abstract

**Background:**

Optimal therapy for colorectal carcinoma (CRC), a frequently diagnosed malignancy, does not exist. Some of colicins and microcins, ribosomally synthesized peptides by gramnegative bacteria, have shown significant biological activity specifically against different cancer cells in vitro and in vivo conditions. The aim of this prospective study was to evaluate natural colicin and microcin production by large intestinal mucosal bacteria in each stage of colorectal neoplasia and in those with a history of colorectal neoplasia.

**Methods:**

A total of 21 patients with non-advanced adenoma (non-a-A; 16/21 with current and 5/21 with history of non-a-A), 20 patients with advanced colorectal adenoma (a-A; 11/20 with current and 9/20 with history of a-A), 22 individuals with CRC (9/22 with current and 13/22 with history of CRC) and 20 controls were enrolled. Mucosal biopsies from the caecum, transverse colon and the rectum were taken during colonoscopy in each individual. Microbiological culture followed. Production of colicins and microcins was evaluated by PCR methods.

**Results:**

A total of 239 mucosal biopsies were taken. Production of colicins and microcins was significantly more frequent in individuals with non-a-A, a-A and CRC compared to controls. No significant difference in colicin and microcin production was found between patients with current and previous non-a-A, a-A and CRC. Significantly more frequent production of colicins was observed in men compared to women at the stage of colorectal carcinoma. A later onset of increased production of microcins during the adenoma-carcinoma sequence has been observed in males compared to females.

**Conclusions:**

Strains isolated from large intestinal mucosa in patients with colorectal neoplasia produce colicins and microcins more frequently compared to controls. Bacteriocin production does not differ between patients with current and previous colorectal neoplasia. Fundamental differences in bacteriocin production have been confirmed between males and females.

## Main text

### Background

Colorectal carcinoma (CRC) is after the lung and prostate cancer the third most frequently diagnosed malignancy in males and the second most common malignancy after the breast cancer in females. There were 1006.000 new CRC diagnoses in males and 794.900 in females in 2018 worldwide. A total of 474,600 CRC related deaths in males and 387,100 in females were reported in 2018 worldwide [1].

The conventional strategies used for treatment of CRC are endoscopy (reserved for early carcinoma up to stage T1sm1), chemotherapy, radiotherapy and surgery. The major disadvantage of chemotherapy, which is the main choice of treatment, is the non-specific mode of action associated with significant toxicity and frequently observed secondary resistance of cancer cells towards chemotherapeutic agents [2, 3].

The human large intestine is a home to approximately 10^14^ microbes and to about 1000 bacterial species [4, 5]. The contribution of large intestinal bacteria to the colorectal carcinogenesis has been shown [6], nevertheless, recent data have also suggested, that intestinal microbiota are able to modulate the effectiveness and toxicity of cancer therapies, especially immunotherapy, which has been introduced within the last decade [5, 7, 8].

Bacteriocins are substances ribosomally synthesized by bacteria. They exhibit antimicrobial activity towards other bacterial species [9–11]. Majority of bacteriocins are extremely potent compared with most of their eukaryotic counterparts, exhibiting antimicrobial activity at nanomolar concentrations [2, 12].

Some of colicins and microcins, bacteriocins produced by gramnegative bacteria, have shown biological activity against different cancer cells in vitro and in vivo conditions [13, 14]. The inhibitory activity of bacteriocins towards cancers cells differs significantly when compared to normal cells, therefore, bacteriocins might become an ideal specific anti-cancer weapon [3, 15].

The aim of this prospective study was to evaluate natural colicin and microcin production by large intestinal mucosal bacteria in each stage of colorectal neoplasia and in those with a history of colorectal neoplasia.

## Methods

A total of 63 patients with colorectal neoplasia and 20 controls were enrolled into the study. Three groups of patients with colorectal neoplasia were included: individuals with non-advanced colorectal adenoma, non-a-A (11 men, 10 women, mean age 63 ± 10; 16/21 with current and 5/21 with history of non-a-A), patients with advanced colorectal adenoma, a-A (13 men, 7 women, mean age 65 ± 9,; 11/20 with current and 9/20 with history of a-A) and individuals with CRC (12 men, 10 women, mean age 70 ± 10,; 9/22 with current and 13/22 with history of CRC). Control group consisted from 7 men and 13 women (mean age 57 ± 14). Patients with colorectal neoplasia were in average risk of developing a sporadic colorectal carcinoma. Advanced colorectal adenoma is defined as an adenoma with low grade dysplasia larger than 10 mm and/or containing high grade dysplasia being of any size and/or adenoma of any size with villous component [16]. Delay between the colonoscopy when mucosal biopsies for subsequent bacteriocin production were taken and the previous colonoscopy when the non-avanced or advanced adenoma had been was removed was 51 ± 24 months (min: 1; max: 91 months) and 48 ± 21 months (min: 5; max 77 months) respectively. Duration between the removal of colorectal carcinoma and colonoscopy with mucosal biopsies for assessement of bacteriocin production was 68 ± 50 months (min: 4; max 164 months).

Among controls, individuals with average risk for colorectal carcinoma, with normal findings on colonoscopy and with negative history of colorectal neoplasia and/or inflammatory bowel disease were included.

Polyethylene glycol was used most frequently for bowel preparation. A total of 40% controls, 62% of patients with a non-advanced adenoma, 50% of individuals with an advanced adenoma and 64% of patients with a colorectal carcinoma were prepared with polyethylene glycol. The remaining patients were prepared with sodium phosphate or sodium picosulfate or sulphate salts.

We used the original methodology reported by our group [17]. Mucosal biopsies were taken in the caecum, transverse colon and the rectum during diagnostic and/or therapeutic colonoscopy. Sterile single forceps were used for each biopsy and each individual sample was put into a tube with a culture medium. Microbiological culture followed. The samples were plated on MacConkey, blood and desoxycholate agar; isolation and identification of bacteria using the VITEK 2 system **(**BioMérieux SA, Marcy l’Etoile, Francie) was performed. Obtained bacterial strains from the *Enterobacteriaceae* family were frozen subsequently.

Production of colicins and microcins was evaluated and investigation of bacteriocin type was carried out: frozen bacterial strains were plated on Petri plates with either 1.2% Trypton Yeast agar or Nutrient agar. After a 48-h culture plates were treated with chloroform vapour for 30 min. Trypton Yeast agar with an indicator strain was added. Indicator strains of *E. coli* K12-ROW*, E. coli* C6 (phi) and *Shigella sonnei* 17 were used for the assessment of bacteriocin production. Bacteriocin production was also confirmed by PCR methods.

Determination of type of bacteriocin was accomplished by PCR methods using specific primers for the detection of colicin and microcin genes [18]. Altogether, 23 individual colicin types (colicins A, B, D, E1-E9, Ia, Ib, Js, K, L, M, N, S4, U, Y, 5/10) and 8 microcin types (mB17, mC7, mE492, mH47, mJ25, mL, mM, mV) were evaluated in this study [19]; see Additional file 1 for details.

Obtained data were tested statistically by means of descriptive statistics and Fisher’s exact test with STATISTICA software, version 13, 2013, Tulsa, OK, USA.

All patients enrolled into the study were adequately informed, supplied and signed an informed consent. The project was approved by the Joint Ethical Committee (Charles University in Praha, Faculty of Medicine at Hradec Kralove & University Teaching Hospital Hradec Kralove) under the number 201107 S54. For all data obtained, all personal identification information was removed in compliance with the Czech laws for protection of confidentiality.

## Results

Twenty controls, 21 patients with non-a-A, 20 individuals with a-A and 22 patients with CRC were included. A total of 239 mucosal biopsies were taken (52 controls, 63 non-a-A, 60 a-A, 64 CRC).

**Fig. 1:**
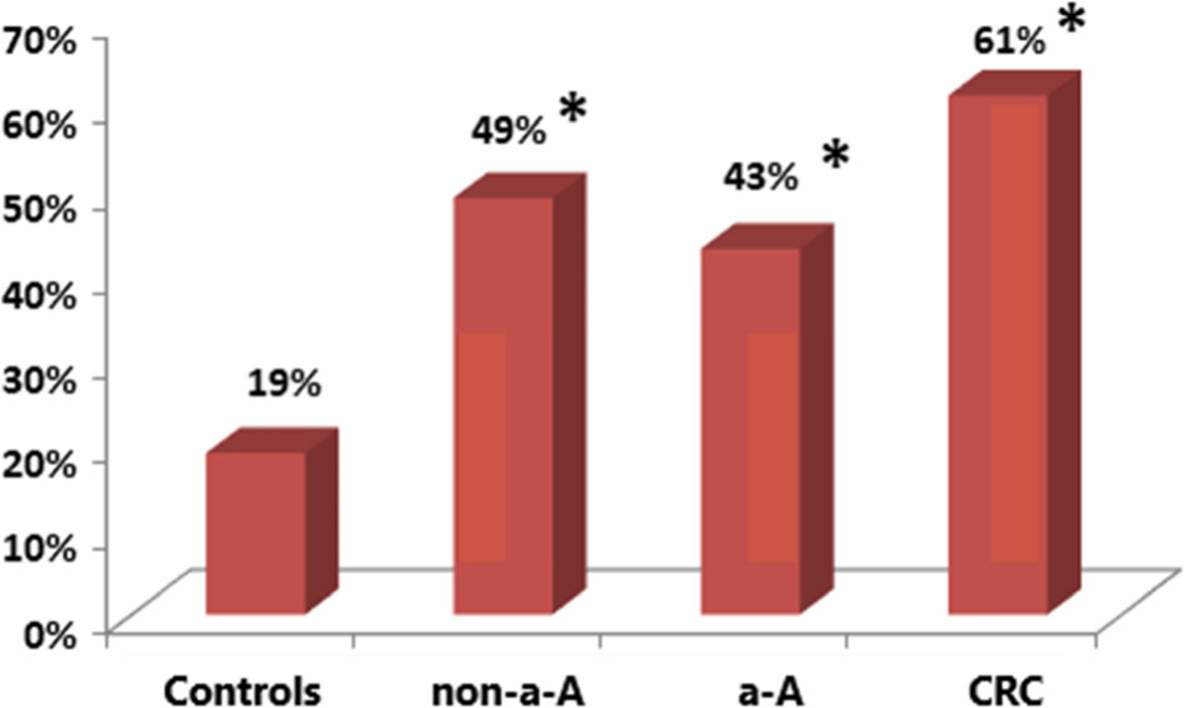
**.** Colicin production in controls, patients with non-advanced adenoma (non-a-A), advanced adenoma (a-A) and colorectal carcinoma (CRC). *: statistically significant difference compared to controls, *p*<0.01.

Colicin producing strains were identified in 19% (10/52) controls, 49% (31/63) non-a-A, 43% (26/60) a-A and in 61% (39/64) CRC; Fig. 1**.**

**Fig. 2:**
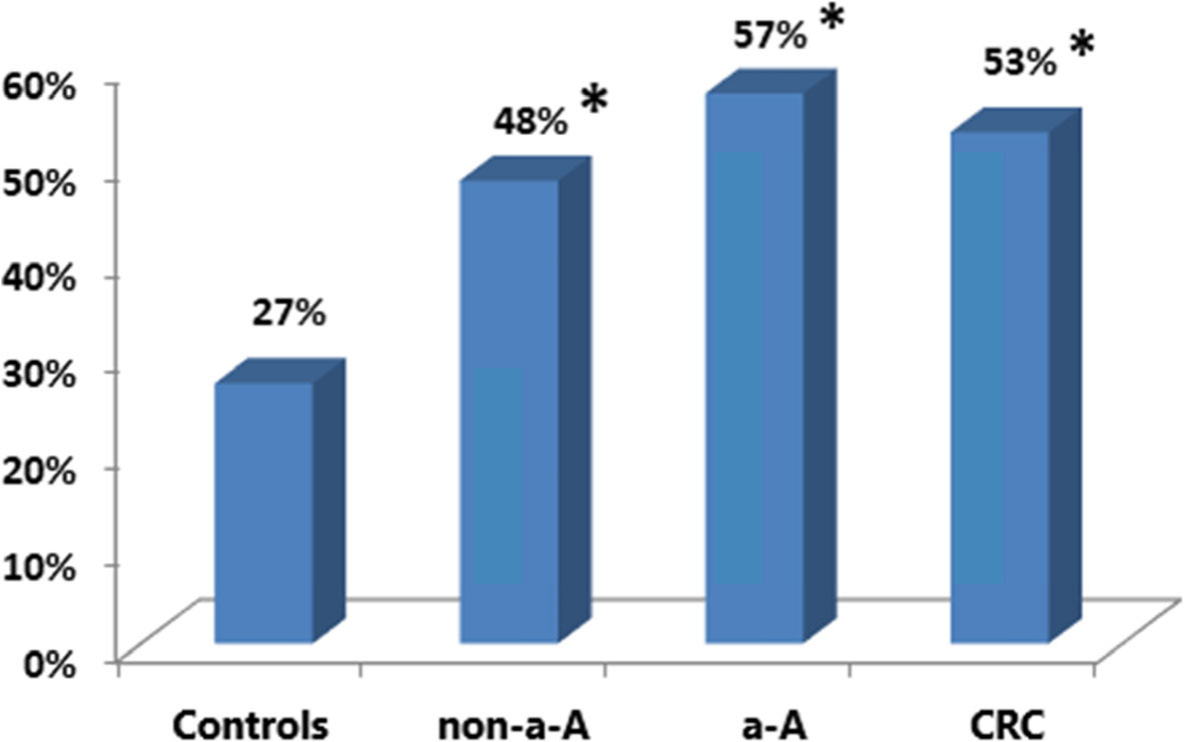
**.** Microcin production in controls, patients with non-a-A, a-A and CRC. *: statistically significant difference compared to controls, *p*<0.05.

Microcin producing strains were found in 27% (14/52) controls, 48% (30/63) non-a-A, 57% (34/60) a-A and in 53% (34/64) CRC; Fig. 2**.**

**Fig. 3:**
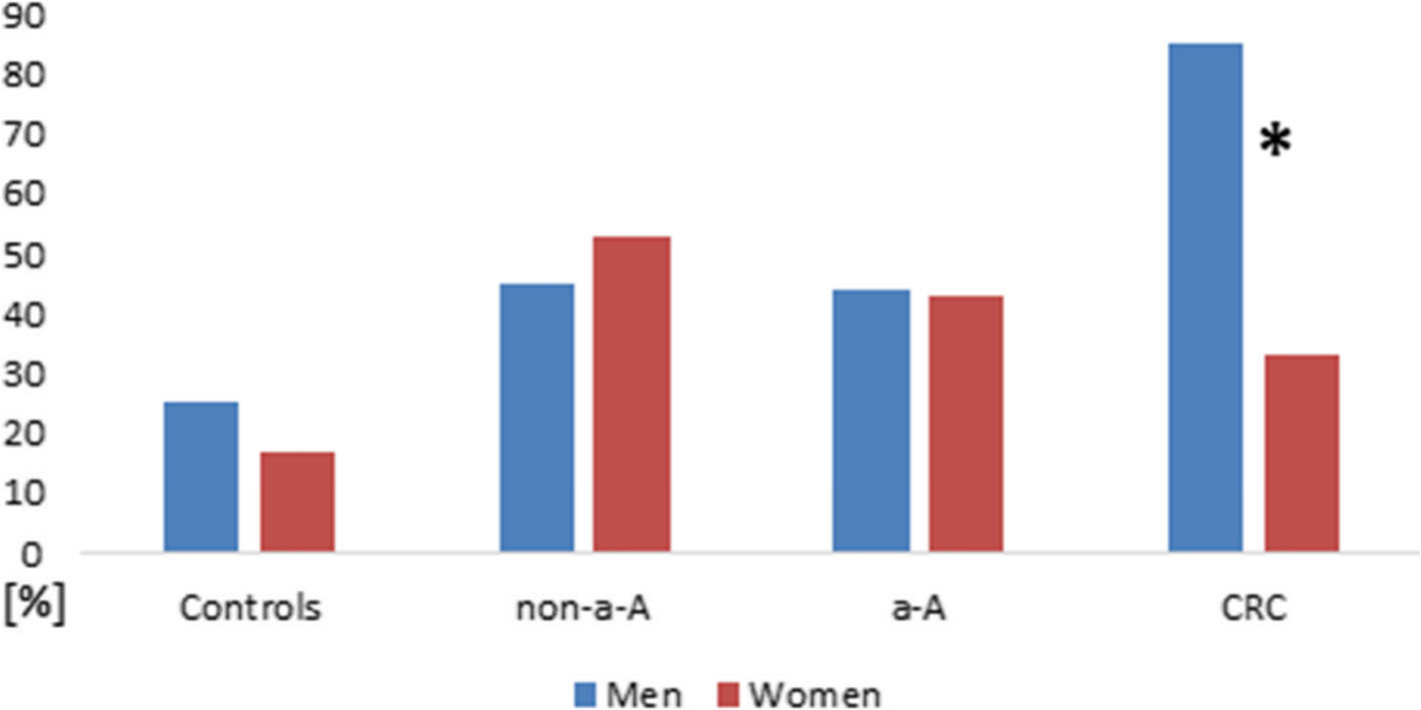
**.** Colicin production in men and women at each stage of colorectal neoplasia. *Statistically significant difference in colicin production between men and women at the stage of CRC; *p*<0.001.

**Fig. 4:**
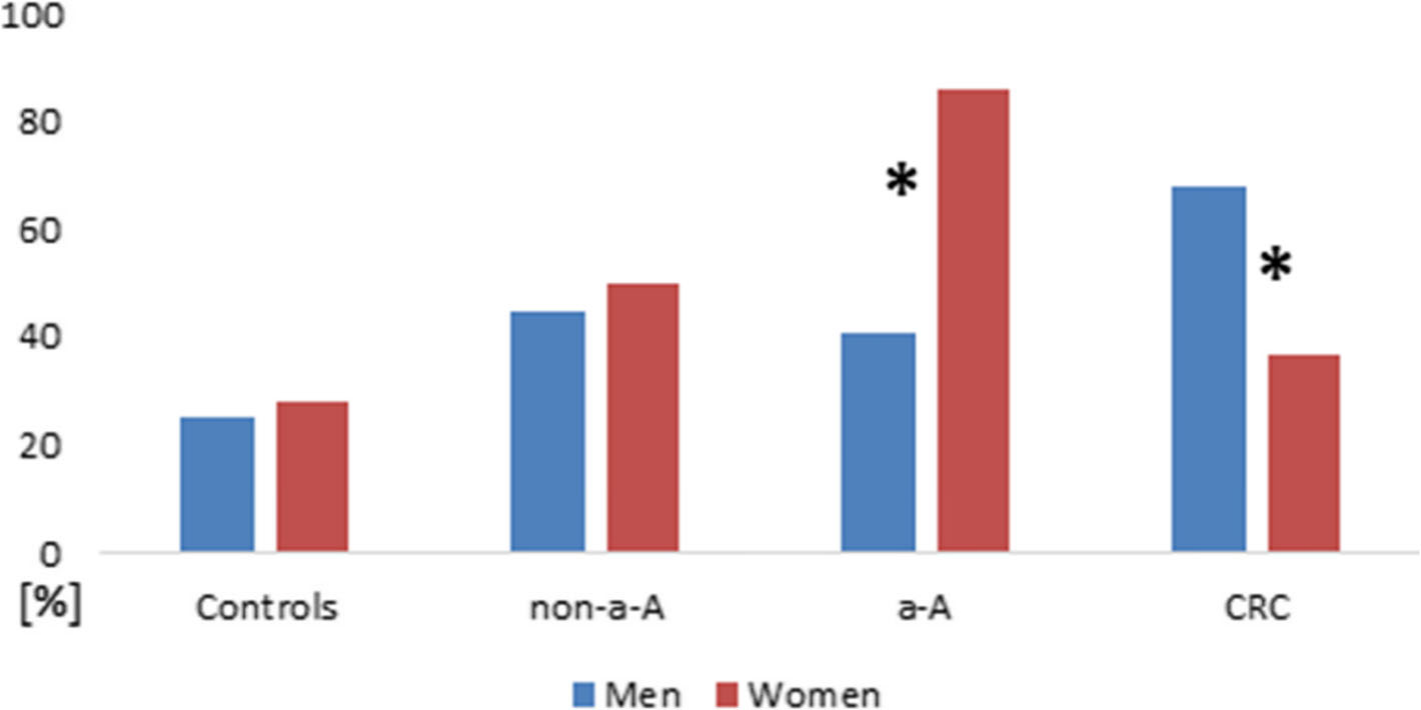
**.** Microcin production in men and women at each stage of colorectal neoplasia. *Statistically significant difference in microcin production between men and women at the stage of a-A; *p*=0.001 and at the stage of CRC; *p*=0.023.

The Figs. 1–4 show proportion of biopsies positive for the production of colicins and microcins.

No significant difference in colicin and microcin production was found between patients with current and previous non-a-A, between individuals with current and previous a-A, between patients with current and previous CRC; *p*>0.05.

Production of colicin and microcin in men and women is shown in Fig. 3 and Fig. 4**.**

**Fig. 5:**
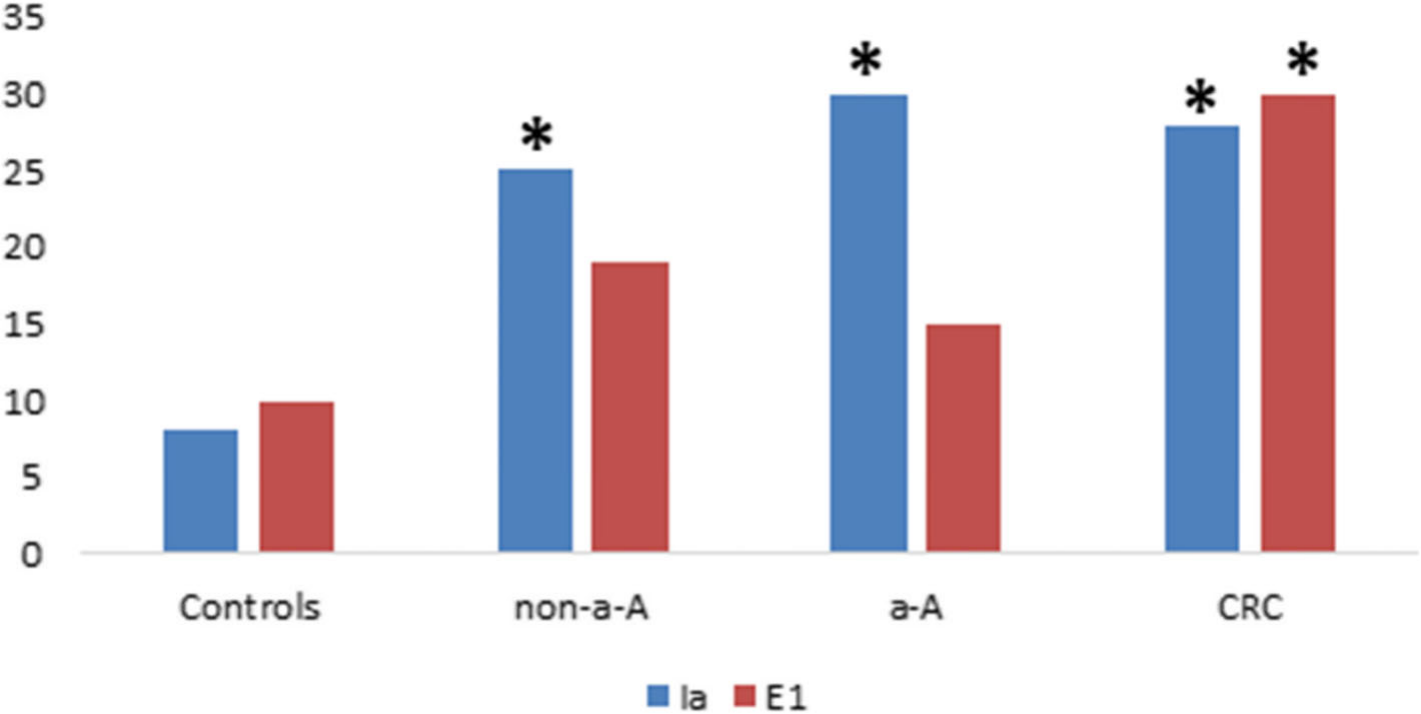
**.** Absolute number of colicin Ia and E1 produced by all strains isolated in each group of colorectal neoplasia and controls. *Statistically significant difference in production of colicin Ia and E1 when compared to controls; *p*<0.02.

Colicin Ia and E1 are the most frequently produced colicins in patients with colorectal neoplasia. Absolute numbers of these colicins in each group tested are shown in Fig. 5**.**

Microcin mH47, microcin mM and microcin mV were the most frequently found microcins across the investigated groups. Microcin mE492 was not produced by any strain.

Our study did not confirm any differences in colicin and microcin production between the caecum, the transverse colon and the rectum in any groups of individuals enrolled in the study; *p*>0.05.

No difference in colicin or microcin production in each group of patients with colorectal neoplasia was found between those prepared for a colonoscopy with polyethylene glycol and those who received other agents for bowel preparation.

The profile of colicin and microcin produced in an individual with current adenoma or carcinoma on colonoscopy was not dependent on the localisation of the neoplasia. The same or a very similar profile of bacteriocin production between all segments was observed.

## Discussion

Our prospective study has shown crucial and novel results in regards to production of colicins and microcins in patients with current and previous colorectal neoplasia. Fundamental differences in bacteriocin production have been confirmed between males and females.

With exception of colicin J_S_ [20], colicins are plasmid-encoded high molecular proteins (>20 kDa) which are produced in a response to stress by *Escherichia coli* and other related gramnegative bacteria from *Enterobacteriaceae* family [2, 21–24]. Four different lethal antimicrobial mechanisms of colicins have been recognized so far: depolarisation of the cytoplasmatic membrane (pore forming colicins) [25], a non-specific DNase activity [26, 27], a highly specific RNase activity [27] and inhibition of murein biosynthesis [28]. The cytotoxic activities of colicins are significantly less understood compared to antimicrobial effects. It is known though, that pore-forming colicins and those exhibiting specific RNase activity have the most toxic effects towards tumour cells [3, 13, 29–31].

E1 colicin belongs to the pore-forming bacteriocins and appears to be a potentially important virulence factor of *E. coli* strains [19, 32]. Our study has documented that colicin E1 (together with another pore-forming colicin Ia) was the most frequent colicin found and its production increased at the stage of CRC significantly. Smarda et al. [29] reported tumour suppressive effect of colicin E1 and E3 (specific RNase activity) on leukemic cells. The effect of colicins was not cell-cycle specific, therefore the authors concluded that the colicins caused necrosis rather than activating apoptotic pathways [29]. Chumchalova and Smarda [13] evaluated in vitro activity of four colicins (A, E1, E3 and U) against one human standard fibroblast line and eleven human tumour-cell lines which carried defined mutations of the p53 Gene. *Colicin* E1 inhibited growth of ten cell lines, and colicin A of all eleven tested cell lines. The colon carcinoma line HT29 was insensitive to colicin E1. Higher fraction of cells in apopotosis after treatment with colicin A and E1 was observed in this study [13]. Further research should aim at evaluation and better understanding of cytotoxic effect of colicins naturaly produced by mucosal intestinal microbiota in individuals with colorectal neoplasia. Effect of these colicins towards adenomatous cell lines [33] and different colon carcinoma cell lines (not only HT29) needs to be assessed. Next step would be evaluation of cytotoxic effect of these colicins in vivo conditions, similarly to the study performed by Tsugu et al. [14]. Subsequently, if clinically and ethically appropriate, prophylactic effect of these colicins could be assessed preferentially in individuals with a high risk of CRC including those suffering from familial adenomatous polyposis or Lynch syndrome.

Microcins, also produced by bacteria from the *Enterobacteriaceae* family, are smaller in size (<10 kDa) when compared to colicins. They are produced in situation of nutrient depletion [2, 34]. Fourteen microcins have been identified so far. They are either plasmid-encoded or chromosomally encoded [35].

Microcin mM, mH47 and mV were the most frequently found microcins across the investigated groups in our study. On contrary, microcin mE492 was not produced by any strain, neither in any patients with advanced colorectal neoplasia. Microcin E492, produced by *Klebsiella pneumoniae*, acts by forming pores [36]. Microcin E492 was shown to display activity against different human cancer lines causing apoptosis at low concentrations and necrosis at higher concentrations [37]. Apoptosis is the desired mechanism for cancer therapy. Lagos et al. [38] have reported that colorectal carcinoma cells are sensitive to microcin mE492 [38]. Probiotic strain *E. coli* Nissle 1917 successfully used in clinical practice, produces microcins mM and mH47 [35, 39]. In 2009, experimental study on animals showed that probiotic *E. coli Nissle 1917* was selectively associated with cancerous cells [40]. As microcins mM and mH47 are closely related to mE492, *E coli Nissle 1917* could become a suitable vector for delivery of mE492 into the tumour [2, 38, 41].

Bures et al. [42] studied production of colicins by luminal bacteria in patients with colorectal carcinoma. The study confirmed that patients with colorectal carcinoma produced significantly less colicins compared with healthy individuals [42]. This was not replicated in our study and the probable explanation is that we studied production of colicins and microcins by mucosal bacteria, which we believe is more relevant in relation to colorectal carcinogenesis. Also different age of the control group might have played a role: 57 years in the current study, 49 in the study performed by Bures at al [42]. Further, bowel preparation which all patients received in the current study has not been carried out in the previous study [42] and this could have influenced the detection of *E. coli* strains.

We are aware, that the bowel cleansing preparation can alter the composition of mucosal adherent microbiota as it was shown by Harrell et al. [43]. Therefore we compared the groups of patients prepared with polyethylene glycol (as an iso-osmotic agent) to all other bowel cleansing agents and we found no differences in colicin or microcin production. Based on this we rather think, that the alteration of composition of intestinal bacteria does not depend on the type of bowel cleansing agent.

Increasing production of colicins and microcins with more advanced neoplasia can not be explained by nutrient deficiency as patients even with advanced colorectal adenoma do not suffer from insufficient supply of nutrients.

The absence of difference in production of colicins and microcins between those with current and previous colorectal neoplasia is fundamental. It confirms, that production of bacteriocins is not a consequence of neoplasia being present. The observation rather acknowledges the comprehensive effect of mucosal bacteria: their contribution to the development of colorectal neoplasia, but also their probable protective effect through production of bacteriocins.

The later onset of increased production of bacteriocins (especially microcins) during the adenoma-carcinoma sequence in men compared to women is also striking. There is no clear explanation for this, and we can only hypothesize that this could play a role among others in the different incidence of CRC in males and females.

Worldwide, there is an immense effort put into the research how to prevent development of colorectal neoplasia and how to optimize treatment of an established colorectal carcinoma. We hope, that bacteriocins could become part of these strategies based on their unique characteristics.

## Conclusions

Strains isolated from large intestinal mucosa in patients with colorectal neoplasia produce colicins and microcins more frequently compared to controls.

Bacteriocin production does not differ between patients with current neoplasia and those with a history of colorectal neoplasia.

A later onset of increased production of microcins during the adenoma-carcinoma sequence has been observed in males compared to females.

Pore-forming colicins Ia and E1 were the most frequently identified colicins. Microcin mM, mH47 and mV belong to the microcins most frequently found across all the groups.

## Supplementary information

**Additional file 1.** Primers used for the detection of colicin genes

## Data Availability

All data generated or analysed during this study are included in the published article and its supplementary information files.

## Abbreviations

a-A: Advanced colorectal adenoma
CRC: Colorectal carcinoma
*E coli*: *Escherichia coli*
non-a-A: Non-advanced colorectal adenoma
PCR: Polymerase Chain Reaction

## References

[1] Ferlay J., Colombet M., Soerjomataram I., Mathers C., Parkin D.M., Piñeros M., Znaor A., Bray F. (2018). Estimating the global cancer incidence and mortality in 2018: GLOBOCAN sources and methods. International Journal of Cancer.

[2] Kaur S, Kaur S (2015). Bacteriocins as potential anticancer agents. Front Pharmacol.

[3] Lancaster LE, Wintermeyer W, Rodnina MV (2007). Colicins and their potential in cancer treatment. Blood Cells Mol Dis.

[4] Vallianou NG, Tzortzatou-Stathopoulou F (2019). Microbiota and cancer: an update. J Chemother.

[5] Chen Danfeng, Wu Jingyi, Jin Duochen, Wang Bangmao, Cao Hailong (2018). Fecal microbiota transplantation in cancer management: Current status and perspectives. International Journal of Cancer.

[6] Heavey PM, Rowland IR (2004). Microbial-gut interactions in health and disease. Gastrointestinal cancer. Best Pract Res Clin Gastroenterol.

[7] Zitvogel L, Ma Y, Raoult D, Kroemer G, Gajewski TF (2018). The microbiome in cancer immunotherapy: diagnostic tools and therapeutic strategies. Science..

[8] Roy S, Trinchieri G (2017). Microbiota: a key orchestrator of cancer therapy. Nat Rev Cancer.

[9] Baindara P, Korpole S, Grover V (2018). Bacteriocins: perspective for the development of novel anticancer drugs. Appl Microbiol Biotechnol.

[10] Braun V, Pilsl H, Gross P (1994). Colicins: structures, modes of action, transfer through membranes, and evolution. Arch Microbiol.

[11] Vasilchenko AS, Valyshev AV (2019). Pore-forming bacteriocins: structural-functional relationships. Arch Microbiol.

[12] Jenssen H, Hamill P, Hancock RE (2006). Peptide antimicrobial agents. Clin Microbiol Rev.

[13] Chumchalová J, Smarda J (2003). Human tumor cells are selectively inhibited by colicins. Folia Microbiol (Praha).

[14] Tsugu H, Onishi H, Fukushima T, Lee S (2006). Anti-tumor activity of de novo designed small globular protein (SGP) in vivo. Anticancer Res.

[15] Cornut G, Fortin C, Soulières D (2008). Antineoplastic properties of bacteriocins: revisiting potential active agents. Am J Clin Oncol.

[16] Mahajan D, Downs-Kelly E, Liu X, Pai RK, Patil DT, Rybicki L (2013). Reproducibility of the villous component and high-grade dysplasia in colorectal adenomas <1 cm: implications for endoscopic surveillance. Am J Surg Pathol.

[17] Kohoutova D, Smajs D, Moravkova P, Cyrany J, Moravkova M, Forstlova M, Cihak M (2014). Escherichia coli strains of phylogenetic group B2 and D and bacteriocin production are associated with advanced colorectal neoplasia. BMC Infect Dis.

[18] Bures J, Smajs D, Kvetina J, Forstl M, Smarda J, Kohoutova D (2011). Bacteriocinogeny in experimental pigs treated with indomethacin and Escherichia coli Nissle. World J Gastroenterol.

[19] Smajs D, Micenkova L, Smarda J, Vrba M, Sevcíkova A, Valisova Z (2010). Bacteriocin synthesis in uropathogenic and commensal Escherichia coli: colicin E1 is a potential virulence factor. BMC Microbiol.

[20] Smajs D, Weinstock GM (2001). Genetic organization of plasmid ColJs, encoding colicin Js activity, immunity, and release genes. J Bacteriol.

[21] Smarda J, Smajs D (1998). Colicins - - exocellular lethal proteins of *Escherichia coli*. Folia Microbiol (Praha).

[22] Cascales E, Buchanan SK, Duché D, Kleanthous C, Lloubès R, Postle K (2007). Colicin biology. Microbiol Mol Biol Rev.

[23] Arnold T, Zeth K, Linke D (2009). Structure and function of colicin S4, a colicin with a duplicated receptor-binding domain. J Biol Chem.

[24] James R, Kleanthous C, Moore GR (1996). The biology of E colicins: paradigms and paradoxes. Microbiology..

[25] Ridley H, Johnson CL, Lakey JH (2010). Interfacial interactions of pore-forming colicins. Adv Exp Med Biol.

[26] Mora L, de Zamaroczy M (2014). In vivo processing of DNase colicins E2 and E7 is required for their import into the cytoplasm of target cells. PLoS One.

[27] Kolade OO, Carr SB, Kühlmann UC, Pommer A, Kleanthous C, Bouchcinsky CA (2002). Structural aspects of the inhibition of DNase and rRNase colicins by their immunity proteins. Biochimie..

[28] Helbig S, Braun V (2011). Mapping functional domains of colicin M. J Bacteriol.

[29] Smarda J, Fialova M, Smarda J (2001). Cytotoxic effects of colicins E1 and E3 on v-myb-transformed chicken monoblasts. Folia Biol (Praha).

[30] Smarda J, Smarda J, Obdrzálek V, Táborský I, Mach J (1978). The cytotoxic and cytocidal effect of colicin E3 on mammalian tissue cells. Folia Microbiol (Praha).

[31] Fuska J, Fusková A, Smarda J, Mach J (1979). Effect of colicin E3 on leukemia cells P388 in vitro. Experientia..

[32] Micenková L, Štaudová B, Bosák J, Mikalová L, Littnerová S, Vrba M (2014). Bacteriocin-encoding genes and ExPEC virulence determinants are associated in human fecal Escherichia coli strains. BMC Microbiol.

[33] Koneczny I, Schulenburg A, Hudec X, Knöfler M, Holzmann K, Piazza G (2015). Autocrine fibroblast growth factor 18 signaling mediates Wnt-dependent stimulation of CD44-positive human colorectal adenoma cells. Mol Carcinog.

[34] Duquesne S, Petit V, Peduzzi J, Rebuffat S (2007). Structural and functional diversity of microcins, gene-encoded antibacterial peptides from *Enterobacteria*. J Mol Microbiol Biotechnol.

[35] Zschüttig A, Zimmermann K, Blom J, Goesmann A, Pöhlmann C, Gunzer F (2012). Identification and characterization of microcin S, a new antibacterial peptide produced by probiotic Escherichia coli G3/10. PLoS One.

[36] Lagos R, Wilkens M, Vergara C, Cecchi X, Monasterio O (1993). Microcin E492 forms ion channels in phospholipid bilayer membrane. FEBS Lett.

[37] Hetz C, Bono MR, Barros LF, Lagos R (2002). Microcin E492, a channel forming bacteriocin from Klebsiella pneumoniae, induces apoptosis in some human cell lines. Proc Natl Acad Sci U S A.

[38] Lagos R, Tello M, Mercado G, García V, Monasterio O (2009). Antibacterial and antitumorigenic properties of microcin E492, a pore-forming bacteriocin. Curr Pharm Biotechnol.

[39] Rembacken BJ, Snelling AM, Hawkey PM, Chalmers DM, Axon AT (1999). Non-pathogenic Escherichia coli versus mesalazine for the treatment of ulcerative colitis: a randomised trial. Lancet.

[40] Brader P, Stritzker J, Riedl CC, Zanzonico P, Cai S, Burnazi EM (2009). Escherichia coli Nissle 1917 facilitates tumor detection by positron emission tomography and optical imaging. Clin Cancer Res.

[41] Maslennikova IL, Kuznetsova MV, Toplak N, Nekrasova IV, Žgur Bertok D, Starčič EM (2018). Estimation of the bacteriocin ColE7 conjugation-based "kill" - "anti-kill" antimicrobial system by real-time PCR, fluorescence staining and bioluminescence assays. Lett Appl Microbiol.

[42] Bures J, Horák V, Fixa B, Komárková O, Zaydlar K, Lonský V (1986). Colicinogeny in colorectal cancer. Neoplasma..

[43] Harrell L, Wang Y, Antonopoulos D, Young V, Lichtenstein L, Huang Y, Hanauer S, Chang E (2012). Standard colonic lavage alters the natural state of mucosal-associated microbiota in the human colon. PLoS One.

