# Supplementary material for: Bacteriocin production by mucosal bacteria in current and previous colorectal neoplasia
Source: BMC Cancer. 2020 Jan 16;20:39. doi: 10.1186/s12885-020-6512-5 (PMC6966821; doi:10.1186/s12885-020-6512-5)
Supplement: Supplementary file 1 — Additional file 1. Primers used for the detection of colicin genes [file 12885_2020_6512_MOESM1_ESM.docx]

**Supplementary material:** Primers used for the detection of colicin genes

| **Colicin** | **Primer** | **Sequention of the primer** | **Lenght of the PCR product** |
| --- | --- | --- | --- |
| **A** | ColA-F | cgtggggaaaagtcatcatc | 475 |
|  | ColA-R | gctttgctctttcctgatgc |  |
| **B** | colicinB-F | aagaaaatgacgagaagacg | 492 |
|  | colicinB-R | gaaagaccaaaggctataagg |  |
| **D** | ColD-F | ctggactgctgctggtgata | 420 |
|  | ColD-R | gaaggtgcgcctactactgc |  |
| **E1** | colicinE1-F | tgtggcatcgggcgagaata | 649 |
|  | colicinE1-R | ctgcttcctgaaaagcctttt |  |
| **E1-1** | cea2F | ggtggaactggaggtagcaa | 357 |
|  | ceaR | acgtcgttgttctgcttcct |  |
| **E2** | ColE2-F | tgatgctgctgcaaaagag | 409 |
|  | ColE2-R | ttcaaagcgttccctaccac |  |
| **E3** | ColE3-F | taagcaggctgcatttgatg | 413 |
|  | ColE3-R | tcggatctggacctttcaac |  |
| **E4** | ColE4-F | gaaggctgcatttgatgct | 409 |
|  | ColE4-R | cggatccggacctttaattt |  |
| **E5** | ColE3-F | taagcaggctgcatttgatg | 430 |
|  | ColE5-R | ttgaattctcgaatcgtcca |  |
| **E6** | ColE6-F | accgaacgtccaggtgtt | 399 |
|  | ColE6-R | tttagcctgtcgctcctgat |  |
| **E7** | ColE7-F | gcattctgccatctgaaat | 431 |
|  | ColE7-R | cttctgcccactttctttcg |  |
| **E8** | ColE3-F | taagcaggctgcatttgatg | 449 |
|  | ColE8-R | gactgattggcttgtcgtga |  |
| **E9** | ColE3-F | taagcaggctgcatttgatg | 418 |
|  | ColE9-R | gacttttctccctccgacct |  |
| **Ia** | ColIa-F | gcatgcaaatgacgctctta | 473 |
|  | ColIa-R | gaggacgccagttctctgtc |  |
| **Ib** | ColIb-F | aacgagtgggtcgatgattc | 464 |
|  | ColIb-R | ccttttctgcgctcgtattc |  |
| **Js** | ColJs-F | tcaaaatgtttgggctcctc | 254 |
|  | ColJs-R | taatctgccctgtcccactg |  |
| **K** | ColK-F | cagaggtcgctgaacatgaa | 469 |
|  | ColK-R | tccgctaaatcctgagcaat |  |
| **L** | Col28b(L)-F | tgcatattgaaagcgtcagc | 449 |
|  | Col28b(L)-R | caggttatcccctctcacca |  |
| **M** | ColM-F | gcttaccacttcgcaaaacc | 429 |
|  | ColM-R | gagcgactctccgataatgc |  |
| **N** | ColN-F | agcttggcgagtatcttgga | 401 |
|  | ColN-R | caacacagccccgaataaac |  |

| **Colicin** | **Primer** | **Sequention of the primer** | **Lenght of the PCR product** |
| --- | --- | --- | --- |
| **S4** | ColS4-F | tatatggcccaactgctggt | 456 |
|  | ColS4-R | cgtaaggacggacacctgtt |  |
| **U** | ColU-F | tgattgctgcgagaaaaatg | 485 |
|  | ColU-R | tctgacagcctctccctgtt |  |
| **Y** | ColY-F | gcaggcagaaaagaacaagg | 477 |
|  | ColY-R | cggacgttatttgccttcat |  |
| **5** | Col5-F | cattggcaaaagcgaaatct | 443 |
|  | Col5-R | tgcaactctggaaacaatcg |  |
| **10** | Col10-F | ggttaccggatttcctggat | 448 |
|  | Col10-R | ttctagatgcttggcccact |  |
| **Fy** | ColFy-Fa | aaattaagcggtgccattgac | 580 |
|  | ColFy-Fa | ttctaattgcgccagacctt |  |

Primers used for the detection of microcin genes

| **Microcin** | **Primer** | **Sequention of the primer** | **Lenght of the PCR product** |
| --- | --- | --- | --- |
| **B17** | mcc B17-F | tcacgccagtctccattaggtgttggcatt | 135 |
|  | mcc B17-R | ttccgccgctgccaccgtttccaccactac |  |
| **C7** | mcc C7-F | cgttcaactgttgcaatgct | 134 |
|  | mcc C7-R | agttgaggggcgtgtaattg |  |
| **E492** | mcc E492-F | gtctctcctgcaccaaaagc | 291 |
|  | mcc E492-R | ttttcagtcatggcgttctg |  |
| **H47** | mcc H47-F | cactttcatcccttcggattg | 227 |
|  | mcc H47-R | agctgaagtcgctggcgcacctcc |  |
| **J25** | mcc J25-F | tcagccatagaaagatataggtgtaccaat | 175 |
|  | mcc J25-R | tgattaagcattttcattttaataaagtgt |  |
| **L** | mcc L-F | ggtaaatgatatatgagagaaataacgtta | 233 |
|  | mcc L-R | tttcgctgagttggaatttcctgctgcatc |  |
| **V** | mcc V-F | cacacacaaaacgggagctgtt | 680 |
|  | mcc V-R | tttcgctgagttggaatttcctgctgcatc |  |
| **M** | micM-4-F | cgtttattagcccgggattt | 166 |
|  | micM-4-R | gcagacgaagaggcacttg |  |
